# The contribution of *FTO* and *UCP*-*1* SNPs to extreme obesity, diabetes and cardiovascular risk in Brazilian individuals

**DOI:** 10.1186/1471-2350-13-101

**Published:** 2012-11-07

**Authors:** Adauto V Ramos, Luciana Bastos-Rodrigues, Bruna A Resende, Eitan Friedman, Luciana Campanha-Versiani, Debora M Miranda, Marta Sarquis, Luiz De Marco

**Affiliations:** 1 Department of Surgery, School of Medicine, Universidade Federal de Minas Gerais, Av. Alfredo Balena 190, Belo Horizonte, 30130-100, Brazil; 2 Hospital Felício Rocho, Belo Horizonte, 30110-068, Brazil; 3 The Susanne Levy Gertner Oncogenetics Unit, Chaim Sheba Medical Center, Tel-Hashomer, 52621, Israel; 4 Department of Pediatrics, Universidade Federal de Minas Gerais, Belo Horizonte, 30130-100, Brazil; 5 Department of Medicine, Universidade Federal de Minas Gerais, Belo Horizonte, 30130-100, Brazil

**Keywords:** FTO, UCP-1, Morbid obesity, Brazilian population, Multiethnic sample

## Abstract

**Background:**

Obesity has become a common human disorder associated with significant morbidity and mortality and adverse effects on quality of life. Sequence variants in two candidate genes, *FTO* and *UCP*-*1*, have been reported to be overrepresented in obese Caucasian population. The association of these genes polymorphisms with the obesity phenotype in a multiethnic group such as the Brazilian population has not been previously reported.

**Methods:**

To assess the putative contribution of both *FTO* and *UCP*-*1* to body mass index (BMI) and cardiovascular risk we genotyped SNPs rs9939609 (*FTO*) and rs6536991, rs22705565 and rs12502572 (*UCP*-*1*) from 126 morbidly obese subjects (BMI 42.9 ± 5.6 kg/m^2^, mean ± SE) and 113 normal-weight ethnically matched controls (BMI 22.6 ± 3.5 kg/m^2^, mean ± SE). Waist circumference, blood pressure, glucose and serum lipids were also measured. Each sample was also genotyped for 40 biallelic short insertion/deletion polymorphism (indels) for ethnic assignment and to estimate the proportion of European, African and Amerindian biogeographical ancestry in the Brazilian population.

**Results:**

Cases did not differ from controls in the proportions of genomic ancestry. The *FTO* SNP rs9939609 and *UCP*-*1* SNP rs6536991 were significantly associated with BMI (p= 0.04 and p<0.0001 respectively). An allele dose dependent tendency was observed for BMI for rs6536991 sample of controls. No other significant associations between any SNP and hypertension, hyperlipidemia and diabetes were noted after correction for BMI and no significant synergistic effect between *FTO* and *UCP*-*1* SNPs with obesity were noted. There was not an association between rs9939609 (FTO) and rs6536991 (UCP-1) in with maximum weight loss after 1 year in 94 obese patients who underwent bariatric surgery.

**Conclusion:**

Our data are consistent with *FTO* rs9939609 and *UCP*-*1* rs6536991 common variants as contributors to obesity in the Brazilian population.

## Background

Obesity, defined by the World Health Organization (WHO) as body mass index (BMI) above 30 kg/m^2^ is an increasingly important clinical and public health challenge in both developed and developing countries and is associated with several co morbidities such as type 2 diabetes, hypertension, cardiovascular diseases and metabolic syndrome [1,2]. More than 400 million adults were obese in 2005, with more than 700 million adults predicted to be obese by 2015 [2]. Although obesity is largely attributed to an imbalance between energy intake and expenditure multiple lines of evidence such as twin and adoption studies [3,4] are consistent with obesity having a contributing genetic component. Thus, in all likelihood obesity is a multifactorial condition due to complex interactions between environmental and genetic factors [2,5,6].

While genes play a role in determining obesity trait, the identification of the genes involved remains elusive for the most part [6,7]. Three independent genome-wide (GWAS) studies reported significant association between obesity (determined by BMI) and common genetic variants in the fat mass and obesity-associated (*FTO*) gene including the SNP rs9939609 [8-10]. This association was also replicated in genetically heterogeneous populations of Caucasians and Asians [11-14], but discordant results have been observed in African populations [15-17]. Furthermore, the differences in risk allele frequencies and linkage disequilibrium structure across ethnicities can provide further insights to refine the association signal and identify the true risk variant.

Of particular interest is the fact that the *FTO* variants do not seem to affect BMI or the risk of obesity in African American [9,15], Chinese Hans [18] or Oceanic populations [19]. The minor allele frequency in these populations is less than half of that reported for populations of European descent and the patterns of linkage disequilibrium are also distinct.

In addition, many association studies were also performed in various populations to elucidate the potential contribution of *UCP1* polymorphisms to various obesity phenotypes [20,21]. Uncoupling protein 1 (UCP1) is abundant in brown adipose tissue, and dissipates energy through heat. Energy expenditure is a complex trait comprising metabolic rate at rest, and physical activity, diet-induced and adaptive thermogenesis [22]. The contribution of the thermoregulatory mechanisms to body weight regulation appears to be critical in homeothermic animals [23]. As such *UCP1* is candidate gene for obesity [24-26].

The association of both *FTO* and *UCP*-*1* polymorphisms with the obesity phenotype in the Brazilian population has not been reported, and that was the aim of the present study.

## Methods

### Participants

We recruited 126 obese patients with mean BMI of 42.9 ± 5.6 kg/m^2^, mean ± SE) from the Bariatric Surgical Clinics of Hospital Felicio Rocho and 113 non-obese controls from General Endocrine Clinic (Hospital Felício Rocho) with mean BMI of 22.6 ± 3.5 kg/m^2^, mean ± SE). Obesity was defined as BMI ≥ 30 kg/m^2^ and non-obese was defined as BMI < 30kg/m^2^. Height and weight were measured with participants dressed in lightweight clothing without shoes. Waist circumference, blood pressure, glucose and serum lipids were also measured. Waist circumference, blood pressure, glucose and serum lipids were also measured. All patients were followed up by the staff of these clinics and data on medication use and diagnosis were considered for enrollment in the study as well as to define disease status.

We genotyped one SNP of the *FTO* gene and three of the *UCP*-*1* gene. The weight of 94 obese patients was obtained one year after bariatric surgery. All participants signed a written informed consent, and the study was approved by the Ethics Committee of the Universidade Federal de Minas Gerais.

### Genotyping

Genomic DNA extraction was performed from whole blood using a standard protocol. The *FTO* rs9939609 and *UCP*-*1* rs6536991, rs2270565 and rs12502572 were genotyped by allelic discrimination Taqman assays (Applied Biosystems, Foster City, CA). PCR was performed in 96-well format in a total of 10μl reaction volume using 10ng of genomic DNA and FAM/VIC dye labeled allelic probes with the Taqman Universal Fast Master mix and subjected to 95°C for 15 min, and 40–50 cycles of 95°C for 15 sec and 60°C for 45 seconds on an ABI 9800 Fast Thermocycler (Applied Biosystems, Foster City, CA). The Taqman assay plates were transferred to ABI 7500 Fast Real Time PCR system in which the fluorescence intensity in each well of the plate was recorded and genotypes were analyzed using Sequence Detection Software 1.3. Genotyping quality control procedures included genotyping 10% duplicates for accuracy checking and inclusion of both positive and non-template controls in each 96-well plate. Genotyping success rate was 99.5%. Genotyping accuracy as determined by concordance between duplicates was 100%.

In addition, each sample was independently typed for 40 biallelic short insertion/deletion polymorphisms (indels) to establish the ancestry of our studied sample [27].

### Statistical analyses

Allele and genotype frequencies were compared between case and control groups with the χ2 test using the *Unphased* software program v.3.0.13. Deviation of allele frequency from Hardy-Weinberg equilibrium (HWE) was tested for all SNPs using the *Haploview* software. In addition, linkage disequilibrium (LD) pattern between the three studied SNPs near the *UCP*-*1* was tested also using *Haploview*. One thousand permutation tests were done for the SNPs near the *UCP*-*1*. We performed 1,000 permutations in each test to estimate the global significance of the results for all analyses and to validate the expectation-maximization values. Putative associations with the *FTO* and *UCP*-*1* loci, including suitable adjustment for age, were assessed via regression analysis. In table 1, the p-value was calculated using Mann–Whitney *U* test to compare continuous variables, and chi-square analysis was performed to compare categorical variables. We used the *Structure* software to estimate the proportion of European, African and Amerindian biogeographical ancestry of each group and the *Unphased* software to analyze the polymorphisms and their association with obesity, diabetes, lipids, hypertension and metabolic syndrome. Nonparametric analysis was performed by the *GraphPad Prism* 5 to analyze a significant synergistic effect between *FTO* and *UCP*-*1* SNPs with obesity. Difference in BMI between genotypes was analyzed using a multiple linear regression, with BMI as the dependent variable and genotype as the independent variable, and with gender as covariate for BMI. Statistical significance was taken at a *p*- value <0.05 for all comparisons.

**Table 1:** Clinical characterization of obese and control subjects*

**Anthropometric measure**	**Obese (n=126)**	**Control (n=113)**	***P value***
Age	40.3 ± 12.7	50.0 ± 17.3	<0.0001
Gender (M/F)	21/105	20/93	0.355
Height (m)	1.63 ± 0.09	1.60 ± 0.09	0.038
Weight (kg)	115.0 ± 21.4	58.3 ± 10.4	<0.0001
BMI	42.9 ± 5.6	22.6 ± 3.5	<0.0001
Waist circumference (cm)	119 ± 13	79 ± 12	<0.0001
Dislipidemia (%)	54.9	46	0.631
HDL (mg/dl)	51 ± 12	47 ± 15	0.0248
LDL (mg/dl)	121 ± 35	125 ± 34	0.3355
Triglycerides (mg/dl)	151 ± 91	134 ± 65	0.3075
Diabetes (%)	17.2	11.4	<0.0001
Glucose (mg/dl)	105 ± 42	89 ± 21	<0.0001
Hypertension (%)	40.9	21.2	0.005
Systolic blood pressure (mmHg)	136 ± 14	131 ± 18	0.0016
Diastolic blood pressure (mmHg)	87 ± 9	82 ± 9	<0.0001

*Mann–*Whitney U test* and chi-square analysis.

## Results

Clinical characteristics of the 239 participants in this study are shown in Table 1. The study sample consisted of 239 Brazilian individuals, aged 18–72 years old. Patients were 126 severely obese subjects (BMI 42.9 ± 5.6 kg/m^2^) with a mean age of 40.3 ± 12.7 years (mean ± SE) (range 18–71 years). Controls were 113 normal-weight (or somewhat overweight) subjects (BMI 22.6 ± 3.5 kg/m^2^) with a mean age of 50.0 ± 17.3 years old (mean ± SE) (range 18–72 years).

Genotyping was performed for one *FTO* SNP (rs9939609) and for three *UCP*-*1* SNPs (rs6536991, rs2270565 and rs12502572). All SNPs were in Hardy-Weinberg equilibrium (*P*-value 0.88, 0.12, 0.35 and 0.49 for rs9939609, rs6536991, rs2270565 and rs12502572 respectively). Table 2 shows the position and Hardy-Weinberg equilibrium of SNPs. The calculated power estimate for the rs2270565 and rs12502572 was 49.4% and 61.0%, respectively.

**Table 2:** Position and Hardy-Weinberg equilibrium of SNPs

**SNP name**	**Chromosome**	**Chromosome position**	**Gene**	**SNP Type**	**Ancestral allele**	Hardy-Weinberg p value **
rs6536991^*^	4	141481581	UCP1	Intron	C	0.12
*rs2270565*	4	141483471	UCP1	Missense mutation	T	0.35
*rs12502572*	4	141485134	UCP1	Intron	A	0.49
rs9939609^*^	16	53820686	FTO	Intron	A	0.88

*tag SNPs; ** HWpval: HWE *P* value’. *X*^2^ tests.

The minor allele frequencies (MAFs) observed in this study (0.435, 0.383, 0.056 and 0.480 for rs9939609, rs6536991, rs2270565 and rs12502572, respectively) were close to those in dbSNP (http://www.ncbi.nlm.nih.gov/snp) population (0.355, 0.319, 0.073 and 0.467, respectively).

**Figure 1:**
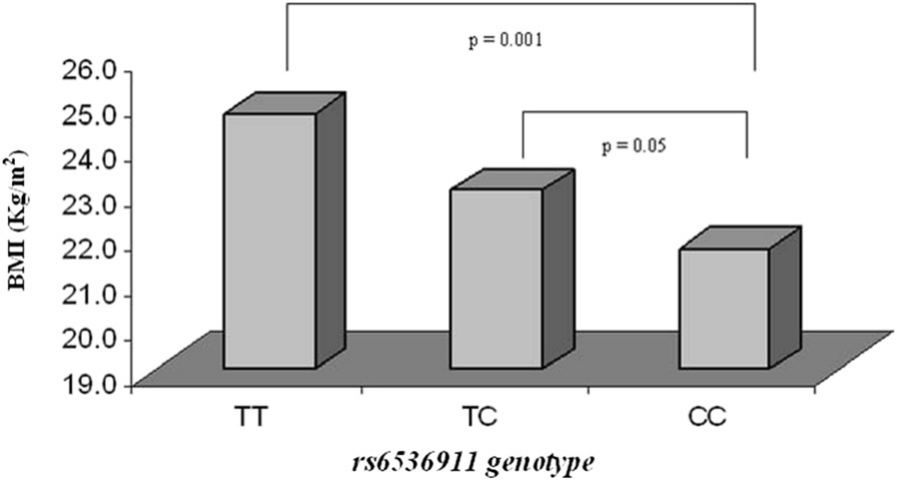
Association between rs6536991 and BMI in control group.

As shown in Table 3, the *FTO* SNP rs9939609 and *UCP*-*1* SNP rs6536991 demonstrated a statistically significant association with the obesity phenotype as measured by BMI (p= 0.04 and p< 0.0001 respectively). However two *UCP*-*1* SNPs rs2270565 and rs12502572 were not associated with the obesity phenotype (p= 0.25 and p= 0.35 respectively). An allele dose-dependent tendency was observed for BMI just for rs6536991 sample of controls (Figure 1). Pairwise LD demonstrated one LD block in the UCP-1 gene comprising two SNPs (rs2270565 and 12502572, D_ = 1.0, LOD 5.29, r-squared 0.082). There was also a moderate LD in UCP-1 rs6536991 and rs12502572 polymorphisms (D_ = 0.781, LOD 37.81, r-squared 0.525) as well as between the polymorphisms rs6536991 and rs2270565 (D_ = 0.637, LOD 0.6, r-squared 0.015).Clinical characteristics of the studied population by *FTO* rs9939609 and *UCP*-*1* rs6536991 genotypes are shown in Tables 4 and 5, respectively.

**Table 3:** **Association between *FTO ***(**rs9939609**), ***UCP***-***1 ***(**rs6536991, rs2270565 and rs12502572**) **with obesity and Hardy**-**Weinberg equilibrium **(**HWE**)

**Genotype**	**Patients**		**Controls**		***P***	**OR**	**95% CI**	**HWE**
	**n**	**%**	**n**	**%**				
rs 9939609								
TT	34	27	41	36	0.04	1	-	0.88
TA	60	48	57	50		1.27	0.71-2.27	
AA	32	25	15	13		2.57	1.2-3.52	
Allele								
T	128	51	139	62	0.02	1	-	
A	124	49	87	38		1.55	1.07-2.23	
rs6536991								
TT	10	8	17	15	<0.0001	1	-	0.12
TC	87	69	42	37		3.52	1.48-8.35	
CC	29	23	54	48		0.91	0.37-2.25	
Allele								
T	107	42	76	34	0.046	1	-	
C	145	58	150	66		0.69	0.47-1.0	
rs2270565								
TT	109	87	104	92	0.25	1	-	0.35
TA	16	13	9	8		1.70	0.72-4.01	
AA	1	1	0	0		2.968e+^007^	1109e+^007^-7943e+^007^	
Allele								
T	234 93	217 96	0.13	1	-			
A	18 7	9 4		1.86	0.82-4.21			
rs12502572								
GG	40 32	46 41	0.35	1	-	0.49		
GA	61 48	47 42		1.49	0.84-2.64			
AA	25 20	20 18		1.44	0.70-2.97			
Allele								
G	141 56	139 62	0.22	1	-			
A	111 44	87 38		1.26	0.87-1.81			

**Table 4:** **Clinical and laboratory data**^**a **^(***FTO *rs9939609 genotype**)

	**Age (years)**	**Weight (Kg)**	**Height (cm)**	**Waist (cm)**	^**b**^**BMI (kg/m**^**2**^**)**	^**c**^**HDL (mg/dl)**	^**d**^**LDL (mg/dl)**	^**e**^**TG (mg/dl)**	^**f**^**SBP (mmHg)**	^**g**^**DBP (mmHg)**	**Fasting Glucose (mg/dl)**
**Obese**											
TT	37.7 ±13.3	115.8±25.2	163.3±10.2	116.5±14.2	43.1±6	52.8±16.1	126.2±33.9	149.9±101.2	137.1±14.7	86.2±7.3	109.9±58.8
	(33.1-42.4)	(107–124.6)	(159.7-166.8)	(111.4-121.5)	(41–45.1)	(46.9-58.6)	(114–138.5)	(113.5-186.4)	(131.9-142.2)	(83.6-88.7)	(88.7-131.1)
TA	42.6±12.6	115.8±19.4	163.2±8	122.0±12.8	43.3±5.7	51.1±10.9	126.2±34	161.0±98.7	135.2±13.9	86.3±8.9	105.4±40.3
	(39.8-45.7)	(110.8-120.8)	(161.1-165.3)	(118.6-125.4)	(41.9-44.8)	(48.2-54)	(117.1-135.3)	(134.8-187.2)	(131.6-138.8)	(84–88.6)	(94.7-116.1)
AA	38.8±12.1	112.7±21.2	163.8±9.2	117.4±12.3	41.8±4.8	49.8±10.1	107.6±33.4	134.3±59.9	136.3±13.4	87.3±9.5	99.0±20
	(34.4-43.2)	(105.1-120.4)	(160.5-167.2)	(112.9-121.8)	(40–43.5)	(46.1-53.4)	(95.6-119.7)	(112.7-155.9)	(131.4-141.1)	(83.9-90.7)	(91.8-106.3)
**Control**											
TT	53.3±17.6	56.4±10.7	160.3±8.9	77.4±11.1	21.9±3.4	46.0±12.3	119.3±33.9	143.9±63.9	130.0±18.5	80.4±9.6	91.1±28.1
	(47.8-58.9)	(53–59.8)	(157.5-163.1)	(73.3-81.4)	(20.8-22.9)	(41.7-50.4)	(107.3-131.3)	(121.2-166.5)	(124.2-135.9)	(77.4-83.4)	(81.3-100.9)
TA	49.8±15.8	60.2±11	160.2±8.2	81.7±12.4	23.4±3.7	48.4±16.1	129.4±33.3	124.5±66.9	131.8±18	81.9±8.8	85.1±13.9
	(45.6-54.0)	(57.2-63)	(158.1-162.4)	(77.9-85.6)	(22.4-24.4)	(43.4-53.5)	(119–139.8)	(103.6-145.3)	(127.11-136.6)	(70.6-84.3)	(80.8-89.4)
AA	41.7±20.2	56.0±5.3	160.6±10.8	72.6±10.4	21.8±2.1	45.9±17.9	126.8±35.4	136.4±64.3	130.3±15.1	82.9±6.5	93.0±18
	(30.5-52.9)	(53–58.9)	(154.6-166.5)	(66–79.2)	(20.6-23)	(35.1-56.7)	(105.4-148.2)	(97.5-175.3)	(121.9-138.6)	(79.3-86.6)	(82.6-103.4)

^a^Data shown as average ± SD and 95% confidence interval of the mean; ^b^BMI, Body Mass Index; ^c^High Density Lipoprotein; ^d^LDL, Low Density Lipoprotein; ^e^TG, Triglycerides; ^f^SBP, Systolic Blood Pressure; ^g^DBP, Diastolic Blood Pressure. TT denotes homozygous carriers of the T allele, TA heterozygous carriers, and AA noncarriers of *FTO.*

**Table 5:** Clinical and laboratory data^a ^(*UCP*-*1 *rs6536991 genotype)

	**Age (years)**	**Weight (Kg)**	**Height (cm)**	**Waist (cm)**	**BMI (kg/m**^**2**^**)**	**HDL (mg/dl)**	**LDL (mg/dl)**	**TG (mg/dl)**	**SBP (mmHg)**	**DBP (mmHg)**	**Fasting Glucose (mg/dl)**
**Obese**											
TT	40.5±9.5	124.2±29.4	164.3±10.1	124.6±17.5	45.6±7.8	47.2±6.2	113.1±30.1	142.1±48.1	131±8.7	83.5±6.7	98.5±15.8
	(33.7-47.3)	(103.2-145.2)	(157.1-171.5)	(112.1-137.1)	(40–51.2)	(42.7-51.7)	(91.6-134.6)	(107.7-176.5)	(124.7-137.3)	(78.7-88.3)	(87.2-109.8)
TC	39.8±12.6	114.6±20.9	163.6±8.8	119.7±12	42.6±5.8	50.6±13.2	119.6±35.7	154.0±96.7	137.4±14.1	87.2±8.7	107.7±48.6
	(37.1-42.5)	(110.2-119.1)	(161.8-165.5)	(117.1-122.3)	(41.4-43.8)	(47.8-53.5)	(111.8-127.4)	(132.8-175.1)	(134.4-140.4)	(85.3-89)	(97.1-118.3)
CC	41.9±14.2	113.0±20	162.4±8.9	116.5±14.7	42.7±4.7	54.4±10.7	129.3±32.1	145.4±85.7	133.4±14.5	85.7±8.9	98.9±23.2
	(36.5-47.3)	(105.4-120.6)	(159–165.8)	(111–122.1)	(40.9-44.5)	(50.1-58.6)	(116.6-142)	(112.2-178.7)	(127.9-138.9)	(82.3-89.1)	(89.9-107.9)
**Control**											
TT	51.2±16.3	64.0±9.7	161.0±6.1	85.8±11.2	24.7±3.4	54.8±15	134.1±39.1	127.5±52.4	135.7±21.7	81.6±6.3	80.0±12.6
	(42.9-59.6)	(59–69)	(157.8-164.2)	(78.6-93)	(22.9-26.4)	(46.1-63.5)	(111.5-156.7)	(97.2-157.8)	(124.5-146.9)	(78.4-84.9)	(72.7-87.2)
TC	52.1±15	58.0±9.7	158.4±8	81.2±17.8	23.0±3.1	42.3±11.4	133.4±35.8	157.6±67.7	134.2±17.8	83.3±9.2	91.1±27.3
	(47.5-56.8)	(55–61.1)	(155.9-160.9)	(77.5-84.9)	(22.1-24)	(38.3-46.2)	(121.6-145.2)	(135.3-179.8)	(128.7-139.8)	(80.4-86.2)	(82.1-100.1)
CC	48.0±19.3	56.6±10.7	161.6±9.8	74.4±11.6	21.7±3.5	49.3±16.4	113.1±25.6	110.4±59.5	126.9±15.7	80.1±9.1	89.3±14.9
	(42.7-53.3)	(53.7-59.5)	(158.9-164.2)	(70.6-78.2)	(20.7-22.6)	(43.8-54.9)	(104.4-121.8)	(90.3-130.5)	(112.7-131.2)	(77.6-82.6)	(84.4-94.2)

^a^Data shown as average ± SD and 95% confidence interval of the mean; ^b^BMI, Body Mass Index; ^c^High Density Lipoprotein; ^d^LDL, Low Density Lipoprotein; ^e^TG, Triglycerides; ^f^SBP, Systolic Blood Pressure; ^g^DBP, Diastolic Blood Pressure. TT denotes homozygous carriers of the T allele, TC heterozygous carriers, and CC noncarriers of *UCP*-*1* rs6536991.

There was a seemingly statistically significant association between rs6536991 with diabetes (p=0.01), rs6536991 with hypertension (p=0.003) and rs6536991 with dyslipidemia (p=0.035) which disappeared after BMI stratification. A borderline association between rs9939609 with diabetes (p=0.05) also disappeared after BMI stratification. We did not find a significant synergistic effect between *FTO* and *UCP*-*1* SNPs with obesity. Difference in BMI between genotypes was analyzed using a multiple linear regression and confirmed the association between *FTO* rs9939609 (p=0.0420) and *UCP*-*1* rs6536991 (p<0.001) genotypes with BMI.

There was not an association between rs9939609 (FTO) and rs6536911 (UCP-1) with maximum weight loss in 94 obese patients one year after bariatric surgery (p=0.410 and p=0.394 respectively).

Using genotyping data for 40 polymorphic indel loci, which form a powerful ancestry informative test battery (27) the proportion of Europeans, Africans and Amerindians were 0.872 ± 0.021, 0.087 ± 0.017 and 0.039 ± 0.092 (mean ± SE), respectively for the cases and 0.928 ± 0.012, 0.037 ± 0.006 and 0.033 ± 0.061, respectively for the controls. The differences in the proportions of genomic ancestries between the two groups were not statistically significant (p = 0.822, p = 0.312 and p= 0.324, respectively).

## Discussion

Gene variants have been reported in association with obesity or obesity-related phenotypes. However, lack of replication has long been a big challenge in these genetic association studies [28]. The association of the *FTO* gene with human obesity is robust in populations of European descendent [8-10]. To date, negative results have involved non-European populations [13,14,16-19]. Although the genetic architecture of the *FTO* locus has not been examined in great detail in these populations evidence is emerging that rs9939609 might be in tight linkage disequilibrium with a casual variant in populations of European descendent. However, this linkage disequilibrium may break down in other ethnic and racial groups suggesting that these population have differences arisen through evolutionary divergence, perhaps as a result of some negative selection pressure against the FTO risk alleles in some African and East Asian population [12-18].

Interestingly, the study by Frayling *et al*. (2007) [8] identified *FTO* through a genome-wide association study for type 2 diabetes. After adjusting for BMI, the association with type 2 diabetes was completely abolished, suggesting that the *FTO*-type 2 diabetes association was mediated through BMI. Subsequently, the association with BMI and obesity was unequivocally replicated in 13 cohorts comprising more the 38000 individuals [29]. The effect of *FTO* SNPs on BMI is modest, with those individuals homozygous for the risk allele weighting, on average, 3 kg more than those homozygous for the protective allele [8]. However, physical activity can attenuate this *FTO* association [30]. BMI-associated SNPs lie within a 47 kilobase (kb) linkage disequilibrium (LD) block encompassing parts of the first two introns as well as exon 2 of the *FTO* gene. Thus, the association signal could be due to correlation between *FTO* intronic SNPs and variation elsewhere in the gene or control elements of other genes [8]. The precise mechanism by which the *FTO* gene leads to obesity development is unclear [31]. The *FTO* gene encodes a 2-oxoglutarate-dependent nucleic acid demethylase that is present in many tissues and is most abundant in the hypothalamus where the control center of energy balance lie [8,31]. Studies in mice showing that Fto mRNA levels are regulated by feeding and fasting have provided a mechanistic link between *FTO* and body weight and energy homeostasis [31]. Cecil *et al*. (2008) [32] demonstrated that a predisposition to obesity does not appear to be involved in the regulation of expenditure but may have a role in the control of food intake and food choice, suggesting a link to a hyperphagic phenotype or a preference for energy-dense foods.

In this report we confirmed the association of the *FTO* variant with BMI in a population of Brazilians with multi-ethnic ancestry. The obese population who was homozygous for the risk allele weighted 3.1kg and 1.3kg/m^2^ more than those homozygous for the protective allele as demonstrated by Frayling *et al*. (2007) [8]. We did not find evidence of association with type 2 diabetes or most of the obesity–related phenotypes in quantitative trait analyses mainly after stratification for BMI as also demonstrated by Frayling *et al*. (2007) [8].

In addition, many studies have looked for many candidate genes to determine genetic factors implicated in the pathogenesis of obesity, related metabolic disorders and diabetes. *UCP1*, which plays a major role in thermogenesis, was suggested to be one of these candidate genes [33]. Uncoupling protein 1 (UCP1), a 32kDa protein located in the inner mitochondrial membrane, is abundant in brown adipose tissue (BAT), in which UCP-1 allows to re-enter the matrix, bypassing the ATP synthase. The usually low proton conductance of the membrane is increased, which results in an acceleration of mitochondrial respiration. The dissipation of the proton electrochemical gradient leads to an uncoupled respiration and heat production, the main function of BAT (34). UCP-1 expression is strongly induced when thermogenesis is required [34]. UCP1 has been reported to play an important role in thermogenesis and energy expenditure and is implicated in the pathogenesis of obesity and metabolic disorders in human [35-37]. The influence of the polymorphism of *UCP1* gene on obesity had been reported in some studies [20,21] while others found no association [38-40].

In our study we investigated the effect of *UCP*-*1* gene SNPs on obesity and obesity related phenotypes among Brazilian people. Our findings show a significant association between the minor allele rs6536991 but not on rs2270565 and rs12502572 with obesity. Again, after BMI stratification, the correlation between rs6536991 and type 2 diabetes, hypertension and dislipidemia disappeared. Interestingly, the effect of the rs6536991 was greater than the effect of *FTO* rs9939609 in our population, with those individuals homozygous for the risk allele weighting were on average, 3kg/m^2^ of BMI more than those homozygous for the protective allele. Taken together we found evidence that rs9939609 in the *FTO* and rs6536991 in the *UCP*-*1* gene increased the risk of obesity but not obesity related phenotypes in the Brazilian population studied. The SNPs rs2270565 and rs12502572 from the *UCP*-*1* were not correlated with obesity and obesity related phenotype.

The reasons for the non effect of the SNPs rs2270565 and rs12502572 could be the relatively small sample size we report or because they were not in linkage disequilibrium compared to rs6536991, which showed association, justifying different behaviors between them. Indeed, a sample size of 445 and 350 people, respectively, would be required to ensure an 80% power. Furthermore, we did not find a significant synergistic effect between *FTO* and *UCP1* SNPs with BMI and no significant correlation with maximum weight loss one year of bariatric surgery. Further studies examining a larger sample size would be necessary to detect this synergistic effect or this association with weight loss one year after bariatric surgery [41].

Brazilians form a very heterogeneous population, which is the result of five centuries of inter-ethnic crosses between peoples from three continents: the European colonizers mainly represented by the Portuguese, African slaves, and the autochthonous Amerindians. Genomic controls help to rule out alternate explanations regarding the influence of racial and ethnic ancestry on this important health outcome [6]. These three groups have admixed to a point which there is very little correlation between skin color and ancestry [42]. Despite the more representative European ancestry, the differences in the proportions of genomics ancestry between the two groups were not significant. These findings bring interesting remarks on the social and genetic epidemiology of obesity.

Some potential limitations should be considered in our study. First, a limited sample size from a single center albeit representative of our population. Second, interaction between lifestyle, physical activity and genes could be a confounding factor and these were not investigated.

Despite the recent success in identifying obesity gene variants using the genome wide association approach, it is well established that such variants do not cause obesity without the individual being exposed to an obesity-promoting environment. Also, only a small fraction of the genetic contribution to obesity has presently been identified [43]. This could be owing to a complex interplay between genetic and environmental factors masking the effect of specific genetic variants.

## Conclusions

In conclusion we identified two SNPs that were associated with a greater BMI in our population. We believe that further studies are warranted, with a greater number of subjects, to examine whether other common variants in the *FTO* and *UCP*-*1* genes could be synergistic in the increased risk for obesity or obesity related phenotypes in the Brazilian and other multiethnic populations.

## Competing interests

The authors declare no conflict of interest.

## Authors’ contributions

AVR and MS recruited subjects and performed laboratory work. LB-R performed laboratory work and data analisis. BAR and LC-V performed laboratory work. LD, EF and DMM conceived and designed the study as well as performed data analysis. All authors read and approved the final manuscript.

## Pre-publication history

The pre-publication history for this paper can be accessed here:

http://www.biomedcentral.com/1471-2350/13/101/prepub
